# Urinary Output as a Predictor of Mortality in Cardiogenic Shock: An Explorative Retrospective Analysis

**DOI:** 10.3390/jcm13247706

**Published:** 2024-12-17

**Authors:** Sebastian Markart, Alexander Hermann, Florian Chiari, Gottfried Heinz, Walter S. Speidl, Max Lenz, Christian Hengstenberg, Peter Schellongowski, Thomas Staudinger, Robert Zilberszac

**Affiliations:** 1 Department of Cardiology, Medical University of Vienna, 1090 Vienna, Austriagottfried.heinz@meduniwien.ac.at (G.H.); walter.speidl@meduniwien.ac.at (W.S.S.); max.lenz@meduniwien.ac.at (M.L.); christian.hengstenberg@meduniwien.ac.at (C.H.); 2 Department of Internal Medicine I, Medical University of Vienna, 1090 Vienna, Austria; alexander.hermann@meduniwien.ac.at (A.H.); peter.schellongowski@meduniwien.ac.at (P.S.); thomas.staudinger@meduniwien.ac.at (T.S.)

**Keywords:** cardiogenic shock, urinary output, prognostication

## Abstract

**Background/Objectives**: Cardiogenic shock (CS) remains a critical condition with high mortality rates despite advances in treatment. This study aimed to evaluate the prognostic significance of urinary output at various time intervals during CS and its effectiveness as a predictor of 30-day mortality, particularly in comparison to the Simplified Acute Physiology Score 3 (SAPS 3). **Methods**: We conducted a retrospective analysis of 96 patients diagnosed with CS, assessing urinary output at different intervals (0–6 h, 6–12 h, 12–24 h, and 0–24 h) as potential predictors of 30-day mortality. SAPS 3 was calculated for all patients, and its predictive value was compared to that of urinary output using both univariate and multivariate analyses. Additional analyses included ROC curve assessment and Kaplan–Meier survival analysis. **Results**: Urinary output at 6–12 h was significantly associated with 30-day mortality in univariate analysis. Area under the receiver operating characteristic curves (AUROCs) for urinary output at 0–6 h, 6–12 h, and 12–24 h was 0.61 (*p* = 0.07), 0.63 (*p* = 0.04), and 0.61 (*p* = 0.08), respectively. These AUROCs did not differ significantly between the three urinary output parameters. Regarding the cumulative urinary output of 0–24 h, the most pronounced impact was observed in patients producing less than 0.5 mL/kg/h. In multivariate analysis, when combined with SAPS 3, the predictive power of urinary output diminished. SAPS 3 alone demonstrated significant predictive value with an AUROC of 0.77 (*p* < 0.001). **Conclusions**: While early urinary output is a valuable predictor of 30-day mortality in patients with CS, its prognostic strength is limited when considered alongside comprehensive risk assessments like SAPS 3. These findings suggest that a multifaceted approach, incorporating both early and comprehensive indicators, is essential for accurately predicting outcomes in CS patients.

## 1. Introduction

Cardiogenic shock (CS) frequently leads to compromised renal function, often manifesting as cardiorenal syndrome type 1 [1,2,3]. This impairment arises primarily from two factors: reduced cardiac output (CO), resulting in arterial perfusion deficits, and venous congestion due to elevated central venous pressure (CVP), which extends its effects to the kidneys. The latter is particularly critical, as it overwhelms the autoregulatory mechanisms that typically maintain adequate renal perfusion through adjustments in arteriolar tone [4]. Additionally, neurohormonal responses and vasoconstriction within the renal vasculature further decrease perfusion pressure, potentially leading to ischemia and tubular necrosis [5]. Consequently, urinary output diminishes, renal congestion increases, cytokine release is elevated, and fluid retention worsens, all of which negatively impact cardiac function [1,3,4]. Uremia and electrolyte imbalances further exacerbate cardiac depression and may contribute to arrhythmias [3].

Various clinical interventions, such as mechanical ventilation, positive pressure ventilation, and the use of mechanical circulatory support (MCS) devices, can further compromise renal perfusion [3]. The administration of catecholamines may also aggravate renal blood flow, while the role of contrast agents during percutaneous coronary intervention (PCI) remains debated [6,7,8].

Reduced urinary output serves as a reliable marker of renal tissue hypoperfusion and partially reflects microcirculatory status [9,10]. It is therefore intuitive to consider urinary output as a predictor of mortality, particularly given that impaired renal function in CS is strongly associated with worse outcomes [11,12,13]. Oliguria, defined as urinary output of less than 30 mL per hour, is a well-established criterion for hypoperfusion in major randomized controlled trials and remains integral to CS definitions [14,15,16,17]. Other definitions include less than 720 mL over 24 h or less than 0.5 mL/kg/hour [6,10,18], with anuria being defined as urinary output below 50 mL for at least 12 h [6].

Beyond urinary output, creatinine levels and estimated glomerular filtration rate (eGFR) are independent predictors of mortality in CS [6,19,20]. While more specific biomarkers such as cystatin C or kidney injury molecule 1 (KIM-1) have been explored, they offer no significant prognostic advantage over creatinine [5,6,21]. However, cystatin C, being independent of factors like age and muscle mass, may be useful in selected cases [6]. Notably, 12–14% of CS patients require renal replacement therapy (RRT), which independently predicts higher mortality rates [22,23].

Despite the established importance of urinary output as a prognostic indicator in CS, an analysis of its predictive value at different time points following the onset of shock has not been conducted in a contemporary, heterogeneous CS cohort. Given the routine availability and ease of monitoring urinary output in intensive care units, understanding the temporal differences in its prognostic value could provide critical insights. Investigating these early phases of CS is particularly valuable, as early and tailored interventions may still improve patient outcomes [19,20,24].

## 2. Materials and Methods

### 2.1. Study Objectives

The primary objective of this retrospective cohort study was to assess the predictive value of urinary output at different intervals (0–6, 6–12, and 12–24 h) after the diagnosis of cardiogenic shock (CS) in relation to 30-day mortality. Secondary objectives were to determine whether urinary output in these intervals provided additional independent information for prognostication when compared to a complex scoring system, the SAPS 3 [25,26], and to put the role of the cumulative urinary output within the first 24 h into perspective. 

### 2.2. Study Design

We conducted a retrospective analysis of patient data from two intensive care units (ICUs) at Vienna General Hospital from January 2017 to December 2019. The study protocol complies with the Declaration of Helsinki and was approved by the ethics committee of the Medical University of Vienna; it was exempted from informed consent requirements owing to its retrospective observational study design.

### 2.3. Study Population

#### 2.3.1. Inclusion Criteria

Inclusion criteria specified that only patients admitted and treated in the specified time frame (January 2017 to December 2019) at ICUs and who met the criteria for cardiogenic shock were included. Cardiogenic shock was defined as follows:

- SBP < 90 mmHg for >30 min or use of catecholamines to maintain a SBP > 90 mmHg;
- Clinical or radiological signs of pulmonary congestion;
- Reduced end-organ perfusion (neurological impairment, cold extremities, oliguria with <30 mL/h, or serum lactate concentration >2 mmol/L).

#### 2.3.2. Exclusion Criteria

Patients were excluded based on the following:

- Under 18 years of age;
- Pregnancy;
- Treated for suicidal/autoaggressive actions;
- Treated for intoxication;
- Absence of complete medical records;
- Duration of ICU stay < 24 h;
- Admission transfers from other hospitals without initial ICU admission data;
- Existence of any directive for terminal care at or before ICU admission.

### 2.4. Data Collection

Data were extracted from electronic health records of patients admitted to the two participating intensive care units. Key demographic information, including age, gender, and clinical metrics, such as SAPS-3 at ICU admission, lactate levels, and creatinine levels, were recorded. Urinary output (mL/h) was measured at three specific intervals: 0–6 h (UO0–6), 6–12 h (UO6–12), and 12–24 h (UO12–24) after ICU admission. The L/A ratio was calculated as the ratio of admission lactate to albumin levels, serving as a marker of metabolic stress. Lactate clearance was calculated as the percentage reduction in lactate levels over a 24 h period. This was determined using the following formula: the difference between the admission lactate level and the lactate level at 24 h was divided by the admission lactate level, and the result was then multiplied by 100.

### 2.5. Statistical Methods

#### 2.5.1. Descriptive Statistics

Patient characteristics were summarized for the entire cohort and stratified by 30-day mortality status. Urinary output parameters and other potential predictors were presented similarly. Nominal variables were described using absolute and relative frequencies, while continuous variables were summarized using medians and interquartile ranges. They were compared using chi-square tests (categorial variables) and Mann–Whitney U tests (continuous variables). 

#### 2.5.2. Inferential Statistics 

Univariate logistic regression models were constructed for urinary output parameters and SAPS 3 to assess their association with 30-day mortality, yielding odds ratios (ORs) with confidence intervals (CIs). Receiver operating characteristic (ROC) curves and area under the curve (AUC) values were generated for comparison. A multivariate logistic regression model combining UO0–6, UO6–12, and UO12–24 was also developed, with the resulting probabilities used to form a combined ROC curve. Because of outliners and absent normal distribution urinary output parameters, lactate values (including L/A ratio) and creatinine levels were log-transformed for all regression models. 

#### 2.5.3. Primary Objective 

The AUCs for the three urinary output parameters (UO0–6, UO6–12, and UO12–24) were compared using DeLong’s test to detect significant differences. The significance threshold was adjusted using the Bonferroni–Holm method, yielding adjusted α levels of 0.016, 0.025, and 0.05.

#### 2.5.4. Secondary Objectives 

Each urinary output parameter was combined with SAPS 3 in a multivariate logistic regression model to determine if urinary output provided independent prognostic information beyond SAPS 3. Area under the receiver operating characteristic curves (AUROCs) were generated from these models to assess the combined predictive value. Due to the limited number of events, no more than three variables were included in any multivariate model. Additionally, a Kaplan–Meier survival analysis was conducted to evaluate the predictive value of absolute urinary output in mL/kg/h after 24 h for 30-day mortality.

#### 2.5.5. Multiple Testing

For the primary objective, DeLong’s test was conducted three times, with the two-sided significance level adjusted according to the Bonferroni–Holm method. No adjustments were made for other analyses, which were considered descriptive or exploratory.

#### 2.5.6. Statistical Software

Data analysis was conducted using IBM SPSS Statistics Version 29 (IBM, Armonk, NY, USA) and MedCalc Statistical Software Version 22 (MedCalc Software Ltd., Ostend, Belgium).

## 3. Results

### 3.1. Patient Characteristics

Of the 847 admissions initially reviewed, 100 were excluded due to insufficient documentation, which included the absence of discharge summaries, surgical or transfer reports, or incomplete monitoring records. These cases primarily involved patients temporarily admitted to the participating ICUs for specific procedures, such as transesophageal echocardiography, cardioversion, thoracostomy, or central venous catheter placement, who were promptly transferred back to their original care facility or did not require further intensive care. In fewer than 10% of these 100 cases, the patients were emergency cases who died shortly after admission, and the available documentation did not allow for a clear retrospective diagnosis of shock. Patients with sufficient documentation were included in the study’s analysis.

An additional 10 patients were excluded due to pregnancy or because they were in a state immediately following intoxication or suicide attempt, with one case involving a coma of unclear etiology. The remaining 737 admissions were evaluated for the presence of shock, specifically CS or septic shock. Of these, 208 patients were identified as having shock upon admission or within 24 h. This group included seventy cases of septic shock, twenty-two hypovolemic, four obstructive, one anaphylactic, and four unclear shocks. Three cases involved mixed shock states, either septic-obstructive or septic-hypovolemic.

Half of the 208 shock patients were diagnosed with CS, the majority of whom were admitted to the cardiology-focused ICU. Eight of these CS patients did not have complete 24 h urinary output data; five of them died within the first 24 h. The final study population consisted of 96 patients with CS, who were included in the statistical analysis. Among these, four had an additional septic component, four had a hypovolemic component, and one had an obstructive component, classifying them as mixed CS cases.

**Figure 1:**
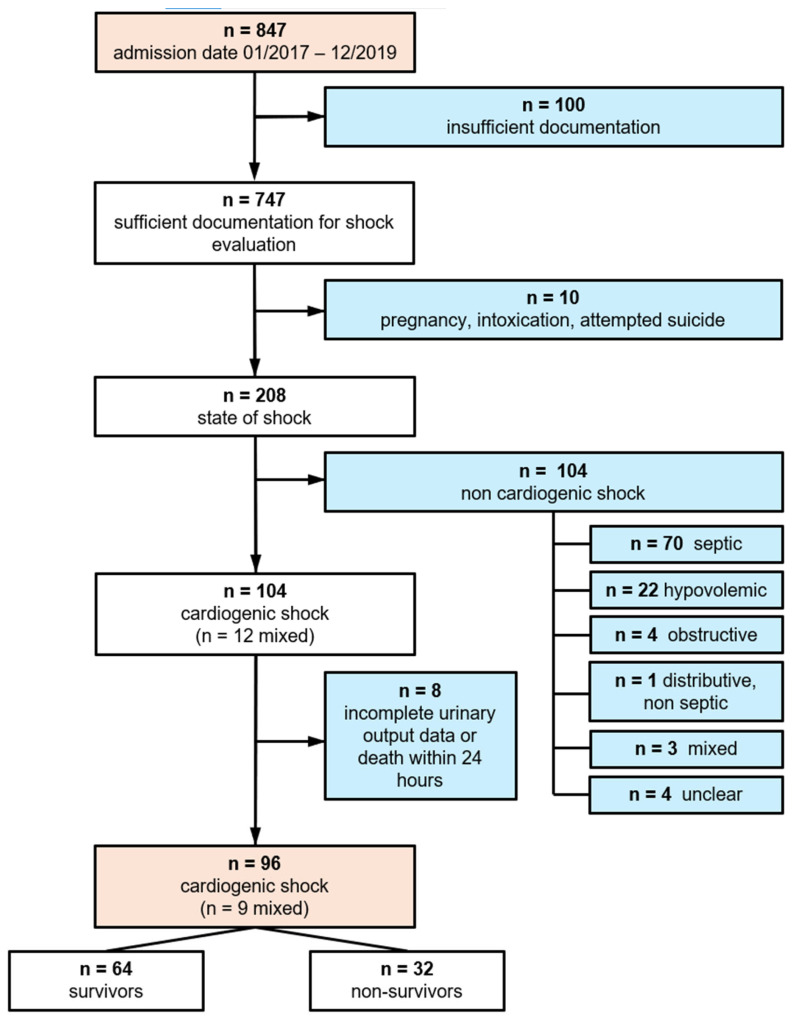
Patient selection flowchart.

This patient selection process is summarized in Figure 1.

Baseline patient characteristics are given in Table 1. 

Of the 96 patients, 32 died within 30 days, and 35 died during their overall hospital stay, resulting in 30-day and hospital mortality rates of 33.3% and 36.5%, respectively. The cohort was predominantly male (71.9%), with a median age of 65 years and a median BMI of 26.8. The age range of the cohort spanned from 18 to 88 years. Median SAPS 3 was 70 points. Over 80% were invasively ventilated and about a quarter received continuous renal replacement therapy (CRRT). Roughly a third underwent extracorporeal membrane oxygenation (ECMO), 28 out of 34 within the first 24 h. More than half of the patients had a history of arterial hypertension and/or coronary artery disease, while approximately 20% had chronic kidney disease (CKD), and 28% had diabetes. Four patients were immunosuppressed due to active malignancy.

Echocardiographic left ventricular function (LVF) data were available for 57 of 96 patients, with 70.2% of these exhibiting severely impaired LVF at ICU admission.

The underlying conditions leading to CS were categorized into key etiologies. Among the ninety-six patients studied, forty-two presented with acute myocardial infarction (AMI), eighteen experienced post-cardiotomy shock, twelve developed CS due to decompensated chronic heart failure, another twelve had valvular cardiomyopathy, five had rhythmogenic causes, and three suffered from acute right heart failure secondary to decompensated chronic pulmonary arterial hypertension. The remaining four patients had other causes of CS.

One-third of all patients had been resuscitated prior to admission, 20 of whom had an AMI. 

Patients who died were older, had higher SAPS 3 scores, were more likely to have been resuscitated before admission, and more frequently received ECMO or CRRT. These patients were more likely to have COPD, CKD, or malignancy as comorbidities. Elective postoperative patients had lower mortality rates. AMI-related CS trended towards an elevated mortality rate of 35.7% versus 31.5%, albeit not reaching statistical significance (*p* = 0.66). 

### 3.2. Study Population Based on Urinary Output and Laboratory and Blood Pressure Values

Table 2 presents the additional parameters collected for the entire study population, with a comparison based on 30-day mortality.

For four patients, lactate values after 24 h were unavailable for analysis. Bilirubin values were missing for eight patients, creatinine for three, and standard bicarbonate and pH values for one patient each. C-reactive protein (CRP), albumin, aspartate aminotransferase (ASAT), and alanine aminotransferase (ALAT) were missing for two patients each. The datasets for the other variables were complete.

Patients who died had lower values across all urinary output parameters and higher SAPS 3. Total fluid intake and volume balance were higher among non-survivors. Admission and 24 h lactate levels were higher in the deceased group, with a lower lactate clearance. Blood pressure values at admission were also lower in these patients compared to survivors.

Standard bicarbonate at admission and the lowest pH within 24 h were reduced in the deceased group. Among the admission laboratory values, bilirubin and creatinine were generally higher in patients who died, while CRP was less elevated in this group.

### 3.3. Urinary Output Parameters and SAPS 3

**Figure 2:**
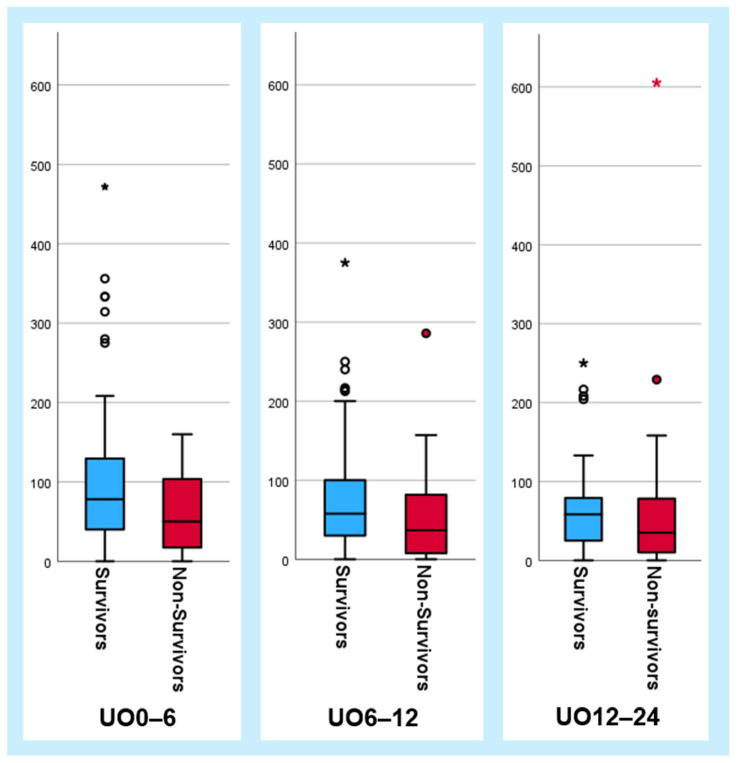
Boxplots illustrating urinary output parameters (mL/h) in relation to 30-day mortality. Outliers are marked as dots, with extreme values represented by asterisks. Abbreviations: UO0–6 = urinary output 0–6 h post-admission; UO6–12 = urinary output 6–12 h post-admission; UO12–24 = urinary output 12–24 h post-admission.

The median urinary output across all analyzed time intervals—UO0–6, UO6–12, UO12–24, and the cumulative output over the first 24 h—differed between survivors and non-survivors with only the values of 6–12 h reaching statistical significance (Table 2). While the interquartile ranges followed this trend, there was substantial overlap between the groups. Boxplots further illustrate the distribution of the three urinary output parameters in relation to 30-day mortality (Figure 2). The lowest values for UO0–6, UO6–12, and UO12–24 were consistently 0 mL/h. Above the maximum values or upper whiskers, several noticeable outliers were observed, primarily among the survivors. Outliers were defined as values exceeding 1.5 times the interquartile range above the third quartile.

Among the survivors, six, five, and four outliers were identified for UO0–6, UO6–12, and UO12–24, respectively, with approximately half representing different patients. The highest hourly urinary outputs for UO0–6, UO6–12, and UO12–24 were 472, 375, and 250 mL/h, respectively, each belonging to different patients. In the non-survivor group, no outliers were observed for UO0–6, with one for UO6–12 at 286 mL/h, and two outliers for UO12–24, the highest being 605 mL/h. The two highest values for UO6–12 and UO12–24 in the non-survivor group belonged to a single patient with hypoxic brain injury following cardiopulmonary resuscitation (CPR) due to AMI, who had a cumulative urinary output of 9315 mL over the first 24 h. The remaining outlier in the non-survivor group for UO12–24 was also a patient who had undergone CPR. In contrast, the majority of outliers in the survivor group had not undergone resuscitation.

The SAPS 3 (maximum possible score 217) range for survivors was 34 to 98 points, while for non-survivors it ranged from 51 to 103 points, with one outlier at 125 points. The median and interquartile range differed significantly (81 (70–90) vs. 66 (55–74)). The outlier in the non-survivor group was a patient who required resuscitation due to acute decompensated heart failure with severe aortic stenosis and also had an active malignant lymphoma. 

Cumulative urinary output after 24 h did correlate with fluid balance (*p* = 0.02) but not with total fluid intake in 24 h (*p* = 0.264).

### 3.4. Univariate Logistic Regression and Survival

Due to the evident outliers and non-normally distributed data, the urinary output parameters were log-transformed for logistic regression analyses. Consequently, the resulting odds ratios (ORs) reflect changes in urinary output by a factor of approximately three (e = 2.718…), which should be considered when interpreting the results. The outcomes of the correlation analyses, ROCs, and AUROCs remain unaffected by this transformation.

The results of the univariate logistic regression indicated a significant association between UO6–12 and SAPS 3 with 30-day mortality (Table 3). The respective ORs were 0.78 (CI 0.60–1.01) for UO0–6, 0.7 (CI 0.52–0.94) for UO6–12, 0.76 (CI 0.58–1.01) for UO12–24, and 1.07 (CI 1.04–1.11) for SAPS 3. The reported *p*-values should be interpreted as exploratory.

**Figure 3:**
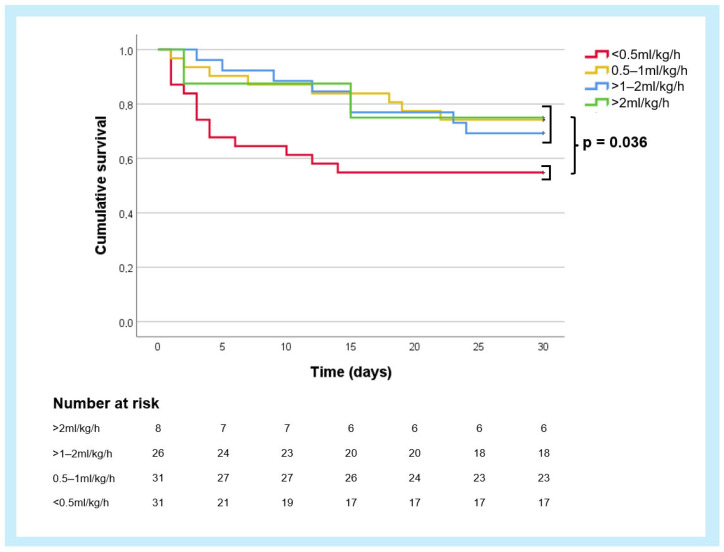
Kaplan–Meier survival curves for cumulative urinary output after 24 h stratified into 4 groups (<0.5 mL/kg/h, 0.5–1 mL/kg/h, 1–2 mL/kg/h, >2 ml/kg/h). Exploratory log-rank test for patients with <0.5 mL/kg/h versus all remaining patients with ≥0.5 ml/kg/h.

In the Kaplan–Meier survival analysis conducted to evaluate the predictive value of absolute urinary output in ml/kg/h after 24 h, the following 30-day survival rates were observed (Figure 3): 54.8% (CI 37.4–72.4%) for patients with urinary output <0.5 mL/kg/h, 74.2% (CI 58.8–89.7%) for those with 0.5–1 mL/kg/h, 69.2% (CI 51.5–87.0%) for 1–2 mL/kg/h, and 75.0% (CI 45.0–100.0%) for >2 mL/kg/h. Although there was a substantial overlap of the survival curves across these groups, patients who produced less than 0.5 mL/kg/h of urine after 24 h had significantly worse survival outcomes compared to the other groups combined (*p* = 0.04). 

### 3.5. Area Under the Receiver Operating Characteristics Curves (AUROCs)

**Figure 4:**
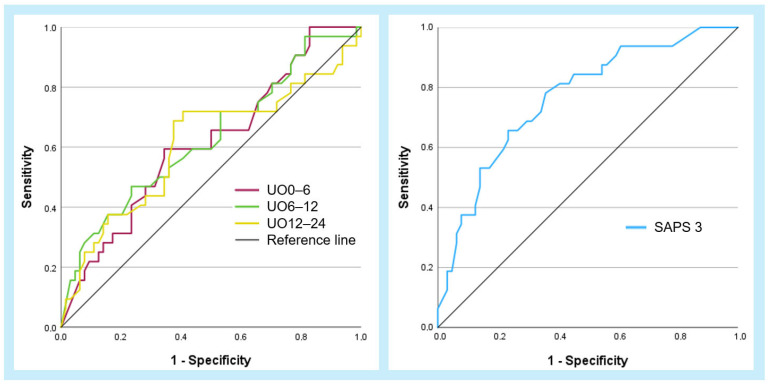
Receiver operating characteristics (ROC) curves for urinary output parameters and SAPS 3 in relation to 30-day mortality. The reference line is set at an area of 0.5. Abbreviations: UO0–6 = urinary output 0–6 h in mL/h; UO6–12 = urinary output 6–12 h in mL/h; UO12–24 = urinary output 12–24 h in mL/h; SAPS 3 = Simplified Acute Physiology Score 3.

The ROC curves for the primary urinary output parameters, as well as SAPS 3, are shown in Figure 4. Each patient’s values are plotted as continuous curves based on their sensitivity and one minus specificity for each parameter in relation to 30-day mortality. The shapes of the curves for UO0–6, UO6–12, and UO12–24 were similar (Figure 4). The curve for SAPS 3 trended more towards the upper left, indicating simultaneously higher sensitivity and specificity. 

SAPS 3 had the largest AUC at 0.77 (CI 0.67–0.87, *p* < 0.001). Among the three urinary output parameters, UO6–12 had the highest AUC at 0.63 (CI 0.51–0.75, *p* = 0.04), followed by UO0–6 with an AUC of 0.61 (CI 0.49–0.73, *p* = 0.07) and UO12–24 with an AUC of 0.61 (CI 0.48–0.74, *p* = 0.08). The reported *p*-values are exploratory (Table 4).

**Figure 5:**
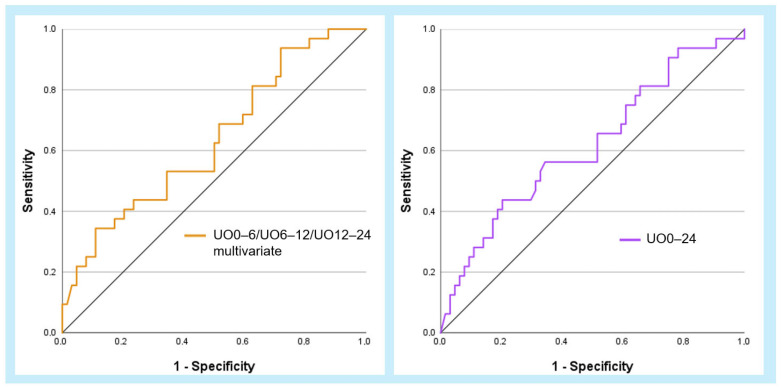
Receiver operating characteristic (ROC) curves for the three primary urinary output parameters in multivariate combination (orange line) and for urinary output 0–24 h (violet line) in relation to 30-day mortality. AUCs are listed in Table 5.

The AUROC of a combined model, which includes UO0–6, UO6–12, and UO12–24, was 0.63 (CI 0.52–0.75), differing by less than one percentage point from the AUC of H6–12 alone. The AUROC of UO0–24, representing the urinary output over the entire first 24 h post-admission in mL/h, was 0.62 (CI 0.48–0.74) (Figure 5, Table 5).

### 3.6. Comparison of AUCs for Urinary Output Across Different Time Intervals in Predicting 30-Day Mortality 

The three DeLong’s tests revealed no significant differences in the AUCs concerning hospital mortality when comparing the UO0–6 and UO6–12 time intervals (*p* = 0.79), the UO0–6 and UO12–24 (*p* = 0.95), as well as the UO6–12 and UO12–24 (*p* = 0.67). The largest absolute difference, or maximum AUC discrepancy, was 0.03 for the UO6–12 versus UO12–24 comparison (Table 6). The significance levels adjusted according to the Bonferroni-Holm method for the smallest, intermediate, and highest *p*-values (α = 0.016, α = 0.025, α = 0.05) were not reached.

### 3.7. Multivariate Analysis of SAPS 3 and Urinary Output in 30-Day Mortality Prediction

The results of four individual multivariate models combining SAPS 3 with each urinary output parameter in relation to 30-day mortality are presented in Table 7. The urinary output parameters were transformed using the natural logarithm. Neither UO0–6 (*p* = 0.29), UO6–12 (*p* = 0.12), UO12–24 (*p* = 0.31), nor cumulative urinary output after 24 h (UO0–24; *p* = 0.28) showed significant results. SAPS 3 achieved a *p*-value of <0.001 in all three models. The reported *p*-values are unadjusted for multiple comparisons and should be considered exploratory. 

The AUC for SAPS 3 combined with UO0–6 was 0.766 (CI 0.668–0.863). SAPS 3 combined with UO6–12 yielded an AUC of 0.779 (CI 0.684–0.875), the AUC for SAPS 3 with UO12–24 was 0.768 (CI 0.669–0.866), and SAPS 3 together with UO0–24 showed an AUC of 0.770 (CI 0.672–0.867). The greatest difference compared to SAPS 3 alone, which had an AUC of 0.765 (CI 0.665–0.865), was observed in the combination of SAPS 3 and UO6–12, with an increase of approximately 1.4 percentage points in area under the curve (Table 8). The reported *p*-values are exploratory. 

SAPS 3 does not contain urinary output as a parameter for renal function, but serum creatinine is included in the score. In an exploratory multivariate model, SAPS 3 was independent from admission creatinine (*p* < 0.001), while creatinine alone did not achieve significance (*p* = 0.67). 

## 4. Discussion

The aim of our study was to evaluate the prognostic significance of urinary output at different time points during cardiogenic shock (CS) and to determine its effectiveness as a predictor of 30-day mortality, particularly in comparison to the SAPS 3 score, a comprehensive risk assessment tool.

Our main findings indicate that urinary output from 6 to 12 h post-admission was associated with survival in CS patients. Concerning the cumulative urinary output within the first 24 h, notably, patients with values less than 0.5 mL/kg/h had significantly poorer survival outcomes. These findings reinforce that reduced urinary output early in CS can indicate increased mortality, underscoring the need for early stabilization and effective therapy in the presence of hypoperfusion signs, even in normotensive patients [18,27]. 

However, when analyzed in a multivariate context alongside the SAPS 3 score, the predictive power of urinary output diminished. While urinary output at 6–12 h post-admission was a significant predictor in univariate analyses, its statistical significance was reduced when the more comprehensive SAPS 3 was included. This suggests that although urinary output is a valuable early indicator, its prognostic strength is lessened when considered alongside a broader risk assessment tool like SAPS 3. 

As early as 1999, the GUSTO-I study [28] underscored the paramount importance of clinical parameters in the prognostic evaluation of cardiogenic shock. The study revealed that the presence of cold, clammy extremities during a myocardial infarction elevated the risk by a factor of 1.68. Moreover, the onset of mental confusion further compounded this risk by an additional 1.68 times, while the development of oligo-anuria was associated with a substantial 2.25-fold increase in risk. Consequently, a persistent urinary output of less than 30 mL per hour was identified as a potential hypoperfusion criterion for the diagnosis of cardiogenic shock, which was linked to a significantly increased 30-day mortality. However, the study did not specify the minimum duration over which this oliguria was considered. 

More recent data from the long-term outcomes of the IABP-SHOCK II study [29] identified initial oliguria of less than 30 mL per hour as an independent predictor of 12-month mortality, and this oliguria threshold (<30 mL/h) had been used as a hypoperfusion criterion for shock diagnosis and inclusion in the IABP-SHOCK II study, which focused exclusively on AMI-CS patients [15]. However, a shorter-term endpoint was not analyzed, and the data collection period was not defined further. 

Regarding 24 h urinary output, an analysis of all admissions to a cardiac intensive care unit found that patients with urinary output under 720 mL within 24 h had twice the in-hospital mortality rate compared to those without hypoperfusion signs [18]. Cardiothoracic postoperative patients and those requiring ECMO therapy were not included. A relationship between 24 h urinary output and 28-day mortality in AMI-CS patients has been established, with urinary output measured continuously rather than dichotomously (oliguria/no oliguria). This approach yielded an odds ratio of 0.999 in the univariate analysis, based on an increase of just 1 mL. Despite the modest effect size, this measure proved significantly more predictive than both admission lactate and the highest lactate level within 24 h (*p* = 0.003 versus *p* = 0.034 versus *p* = 0.025) [30].

A comprehensive analysis of urinary output at various time points during the early phase of cardiogenic shock, or classification based on AUROCs, has not been extensively explored in the literature. In contrast, numerous studies have focused on lactate levels and their temporal changes [31,32,33,34]. Urinary output measured at 6 h intervals has been analyzed, but rather than being treated as a continuous variable, it was classified according to KDIGO stages (stage 1: <0.5 mL/kg/h for ≥6 h; stage 2: <0.5 mL/kg/h for ≥12 h; stage 3: <0.3 mL/kg/h for ≥24 h or anuria). The emphasis was on the minimum duration of oliguria within the first 24 h rather than on changes in urinary output over time. Only stage 1 showed no association with 90-day mortality, while a stricter cutoff of less than 0.3 mL/kg/h for stage 1, as well as stages 2 and 3, were independent predictors of mortality. The patient cohort, largely similar demographically to the population in this study, however, included 81% with ACS etiology and excluded post-cardiotomy patients and rhythmogenic causes [6].

In our univariate analysis, urinary output 6–12 h after admission was predictive of outcomes, and patients with a urinary output of less than 0.5 mL/kg/h within the first 24 h post-admission had significantly worse survival. SAPS 3 emerged as a better predictor overall, despite being multifaceted and thus complex to obtain in situations of clinical ambiguity. This suggests that early in the course of cardiogenic shock, it may be premature to make definitive prognostic assessments based on urinary output alone. The AUC for 24 h urinary output was 0.62, indicating it was not an exceptionally strong predictor either. This could imply that renal failure may manifest later in the disease course or that initial anuria can improve rapidly following normalization of cardiac output, particularly in patients receiving ECMO support [35]. Therefore, early conclusions based solely on urinary output may be limited in their predictive accuracy.

SAPS 3 showed significant predictive power for 30-day mortality in both univariate and multivariate analyses, achieving an AUROC of 0.77 (*p* < 0.001). These results in the mixed CS cohort are consistent with findings from other studies [36,37], where similar outcomes were observed particularly in cohorts focused on AMI patients. In contrast, the CULPRIT-SHOCK registry analysis reported only moderate predictive power for SAPS II, with an AUC of 0.63, specifically in the context of AMI-CS [38].

SAPS 3 had the highest AUROC compared to the urinary output parameters. However, SAPS 3 is a much more comprehensive score aimed at individual prognosis calculation, with numerous subcategories and unequal point distribution, making it complex to use and potentially subject to subjective interpretation due to factors such as the inclusion of the GCS [25,26]. In contrast, urinary output is a singular parameter, but it is simple, early, and ubiquitously accessible.

Strikingly, while including severely ill patients in overt cardiogenic shock, with a high rate of ECMO usage, our study reported a lower mortality rate compared to landmark multicenter trials like ECLS-SHOCK [17] and IABP-SHOCK [15], which reported 30-day mortality rates of 47.8% and 39.7%, respectively. This finding highlights the importance of specialized care in managing cardiogenic shock (CS) patients in centers of excellence. The expertise and resources available in specialized centers may significantly impact patient outcomes, particularly when managing complex scenarios like ECMO [39].

Most landmark trials in the field have traditionally used 30-day mortality as the primary outcome, and we adhered to this standard in our study. However, the seminal DANGER-SHOCK trial [40] opted for a 180-day mortality endpoint due to evidence that late mortality among CS patients can increase significantly over time [41,42,43]. If we had used a similar 180-day endpoint in our study, our findings might have differed, particularly in the context of multivariate analysis. Specifically, H6–12 urinary output would have remained significant alongside SAPS 3, suggesting that this time interval might better capture the evolving nature of patient outcomes over a longer period.

Our study included a low proportion of resuscitated patients—only one-third—compared to recent landmark studies like IABP-SHOCK [15] or ECLS-SHOCK [17]. The inclusion of resuscitated patients in CS cohorts is highly controversial, as resuscitation can introduce a variety of confounding factors that may obscure the true effects of interventions on mortality and other outcomes [44,45]. 

While our study demonstrates the importance of early urinary output monitoring, it also underscores the complexity of CS management and the need for multifaceted assessment strategies. SAPS 3, despite its potentially delayed availability and obvious limitations in acute settings, provided valuable prognostic information, suggesting that a comprehensive approach that includes various different components and both early and later indicators is essential for accurately predicting outcomes in CS patients.

Previous studies have highlighted the strong predictive value of SAPS-3 and APACHE II for short-term mortality in shock patients, alongside lactate as an early, robust prognostic marker. While our study confirmed the predictive utility of SAPS-3, we did not directly compare the prognostic value of lactate alone in patients with differing urinary output. This remains an area for future research to better delineate the interplay between these parameters in predicting outcomes.

The retrospective nature of this study limits the strength of its findings. As is typical in single-center, retrospective studies with relatively small sample sizes, the results are more susceptible to confounding factors and limited by the availability of standardized, parallel measurement times. Thus, these findings should primarily be considered hypothesis-generating. The lack of advanced hemodynamic measurements represents a limitation of our study, reflecting current clinical practice and guideline recommendations [46,47]. SCAI [48] staging was not included as it was published towards the end of our study period and is still undergoing validation across various clinical contexts. 

It might be considered a limitation that no specific subgroup analysis of ECMO patients was performed due to the small sample size, and the limited availability of echocardiographic LVF measurements in our cohort represents a potential constraint of this study.

The cohort included a relatively high proportion of post-cardiotomy shocks (18 of all 96 patients), where an inflammatory or vasodilatory component could have skewed results. The use of diuretics might have obfuscated the measurement of urinary output. However, use of these substances in the initial critical state of hemodynamic collapse is only a rare exception. Patients who died within the first 24 h were excluded from the analysis due to missing urine output data, likely leading to a slight underestimation of the study population’s overall mortality.

## 5. Conclusions

While early urinary output is a valuable predictor of 30-day mortality in patients with CS, its prognostic strength is limited when considered alongside comprehensive risk assessments like the SAPS 3 score. These findings suggest that a multifaceted approach, incorporating both early and comprehensive indicators, is essential for accurately predicting outcomes in CS patients.

## Figures and Tables

**Table 1:** Characteristics of the study population in relation to 30-day mortality. Values are presented as n (%); median [interquartile range]. Abbreviations: HTN = arterial hypertension; AMI = acute myocardial infarction; BMI = body mass index; CAD = coronary artery disease; CKD = chronic kidney disease, any stage; COPD = chronic obstructive pulmonary disease; DM = diabetes mellitus; ECMO = extracorporeal membrane oxygenation; PAD = peripheral/central arterial occlusive disease; CRRT = continuous renal replacement therapy; SAPS 3 = Simplified Acute Physiology Score 3.

	Overall (n = 96)	Non-Survivors (n = 32)	Survivors (n = 64)	*p*-Value
**Demographic Data**				
Male	69 (71.9)	22 (68.8)	47 (73.5)	0.63
Age, years	65 (54–72)	68 (60–75)	64 (51–71)	0.02
BMI, kg/m^2^	26.8 (24.1–30.8)	26.1 (24.5–30.5)	27.0 (23.9–31.0)	0.91
**Cardiogenic Shock**				
AMI	42 (43.8)	15 (46.9)	27 (42.2)	0.66
Cardiac arrest	32 (33.3)	13 (40.6)	19 (29.7)	0.28
**Intensive Care Unit**				
Lenght of stay, days	9 (4–20)	6 (3–12)	11 (6–24)	0.00
Invasive ventilation	80 (83.3)	26 (81.3)	54 (84.4)	0.70
ECMO therapy	34 (35.4)	14 (43.8)	20 (31.3)	0.23
CRRT	25 (26.0)	12 (37.5)	13 (20.3)	0.07
SAPS 3	70 (59–82)	81 (70–90)	66 (55–76)	0.00
**Medical History**				
HTN	51 (53.1)	17 (53.1)	34 (53.1)	1.00
COPD	9 (9.4)	7 (21.9)	2 (3.1)	0.00
CKD	18 (18.8)	6 (18.8)	12 (18.8)	1.00
DM	27 (28.1)	11 (34.4)	16 (25.0)	0.34
CAD	61 (63.5)	21 (65.6)	40 (62.5)	0.76
PAD	15 (15.6)	7 (21.9)	8 (12.5)	0.23
Active malignoma	4 (4.2)	3 (9.4)	1 (1.6)	0.71

**Table 2:** SAPS 3, urinary output, and other parameters in relation to 30-day mortality. Values are presented as median [interquartile range]. Unless otherwise specified, values represent the first measurements after admission. Lactate clearance refers to the first 24 h post-admission. Abbreviations: ALAT = alanine aminotransferase; ASAT = aspartate aminotransferase; CRP = C-reactive protein; UO0–6 = urinary output 0–6 h post-admission; UO6–12 = urinary output 6–12 h post-admission; UO12–24 = urinary output 12–24 h post-admission; UO0–24 = total urinary output after 24 h; L/A ratio = lactate/albumin ratio; MAP = mean arterial pressure; SAPS 3 = Simplified Acute Physiology Score 3.

	Overall (n = 96)	Non-Survivors (n = 32)	Survivors (n = 64)	*p*-Value
SAPS 3 [points]	70 (59–82)	81 (70–90)	66 (55–76)	<0.001
UO0–6 [mL/h]	73 (29–121)	50 (17–104)	78 (40–130)	0.07
UO6–12 [mL/h]	53 (23–93)	37 (8–82)	58 (30–100)	0.04
UO12–24 [mL/h]	48 (21–79)	35 (10–78)	58 (25–79)	0.08
UO0–24 [mL/h]	59 (31–105)	45 (20–84)	63 (40–114)	0.06
Fluid intake total 0–24 h [ml]	5907 (3999–8031)	6348 (5184–9269)	5857 (3629–7263)	0.08
Fluid balance total 0–24 h [ml]	4333 (2139–6107)	5518 (3284–7278)	3636 (1406–5450)	0.01
Lactate at admission [mmol/l]	3.2 (2.1–5.9)	4.0 (2.4–8.1)	2.9 (2.0–5.4)	0.10
Lactate 24 h [mmol/l]	2.0 (1.2–3.1)	3.5 (2.1–7.6)	1.6 (1.1–2.3)	<0.001
Lactate clearance [%]	35.3 (5.4–58.0)	15.0 (-35.7–35.6)	46.7 (27.3–63.9)	<0.001
Bilirubin [mg/dl]	1.05 (0.54–1.87)	1.19 (0.33–2.20)	1.04 (0.60–1.83)	0.56
Creatinine [mg/dl]	1.41 (1.12–1.89)	1.74 (1.21–2.74)	1.35 (1.07–1.71)	0.02
CRP [mg/dl]	1.84 (0.49–9.20)	0.94 (0.43–9.67)	2.20 (0.92–8.6)	0.27
ALAT [U/l]	73 (25–222)	73 (29–327)	63 (25–200)	0.58
ASAT [U/l]	159 (64–509)	171 (80–566)	137 (64–432)	0.49
Albumin [g/dl]	2.9 (2.4–3.3)	3.0 (2.5–3.5)	2.8 (2.3–3.3)	0.27
L/A-Ratio	1.27 (0.73–2.13)	1.44 (0.80–2.41)	1.23 (0.69–1.93)	0.24
Lowest pH within 24 h	7.27 (7.18–7.33)	7.20 (7.14–7.30)	7.29 (7.23–7.34)	<0.001
Bicarbonate [mmol/l]	21.3 (18.1–23.4)	19.5 (17.2–22.8)	21.6 (19.0–23.7)	0.04
Systolic blood pressure [mmHg]	103 (93–115)	95 (86–105)	106 (97–123)	<0.001
Diastolic blood pressure [mmHg]	61 (55–69)	58 (53–68)	62 (55–70)	0.19
MAP [mmHg]	77 (66–84)	73 (64–81)	77 (69–86)	0.01

**Table 3:** Univariate regression in relation to 30-day mortality for urinary output parameters and SAPS 3. Urinary output parameters were log-transformed using the natural logarithm. Abbreviations: 95% CI = 95% confidence interval; UO0–6 = urinary output 0–6 h in mL/h; UO6–12 = urinary output 6–12 h in mL/h; UO12–24 = urinary output 12–24 h in mL/h; SAPS 3 = Simplified Acute Physiology Score 3.

	Regression Coefficient	Odds Ratio	95% CI		*p*-Value
			Lower Limit	Upper Limit	
UO0–6	−0.249	0.780	0.602	1.009	0.059
UO6–12	−0.359	0.699	0.522	0.935	0.016
UO12–24	−0.271	0.763	0.576	1.010	0.059
SAPS 3	0.071	1.074	1.036	1.113	<0.001

**Table 4:** AUCs for urinary output parameters and SAPS 3 in relation to 30-day mortality. Abbreviations: 95% CI = 95% confidence interval; AUC = area under the curve; UO0–6 = urinary output 0–6 h in mL/h; UO6–12 = urinary output 6–12 h in mL/h; UO12–24 = urinary output 12–24 h in mL/h; SAPS 3 = Simplified Acute Physiology Score 3.

	AUC	95% CI		*p*-Value
		Lower Limit	Upper Limit	
UO0–6	0.614	0.494	0.733	0.071
UO6–12	0.628	0.507	0.750	0.041
UO12–24	0.609	0.480	0.737	0.084
SAPS 3	0.765	0.665	0.865	<0.001

**Table 5:** AUCs for the three primary urinary output parameters in multivariate combination and for urinary output 0–24 h. Abbreviations: 95% CI = 95% confidence interval; AUC = area under the curve; UO0–6 = urinary output 0–6 h in mL/h; UO6–12 = urinary output 6–12 h in mL/h; UO12–24 = urinary output 12–24 h in mL/h; UO0–24 = total urinary output after 24 h in mL/h.

	AUC	95% CI		*p*-Value
		Lower Limit	Upper Limit	
UO0–6/UO6–12/UO12–24 multivariate	0.633	0.515	0.751	0.034
UO0–24	0.618	0.479	0.740	0.06

**Table 6:** Comparison of AUCs for the three urinary output parameters related to the primary outcome of 30-day mortality using DeLong’s test. Abbreviations: 95% CI = 95% confidence interval; ∆ AUC = difference in area under the curve; UO0–6 = urinary output 0–6 h in mL/h; UO6–12 = urinary output 6–12 h in mL/h; UO12–24 = urinary output 12–24 h in mL/h.

	∆ AUC	95% CI		*p*-Value
		Lower Limit	Upper Limit	
UO0–6 versus UO6–12	0.0149	−0.0926	0.1220	0.786
UO0–6 versus UO12–24	0.0049	−0.1400	0.1500	0.947
UO6–12 versus UO12–24	0.0198	−0.0717	0.1110	0.672

**Table 7:** Multivariate regression (30-day mortality) for SAPS 3 combined with each urinary output parameter. Urinary output parameters were transformed using the natural logarithm. Abbreviations: 95% CI = 95% confidence interval; UO0–6 = urinary output 0–6 h in mL/h; UO6–12 = urinary output 6–12 h in mL/h; UO12–24 = urinary output 12–24 h in mL/h; UO0–24 = total urinary output after 24 h in mL/h; SAPS 3 = Simplified Acute Physiology Score 3.

	Regression Coefficient	Odds Ratio	95% CI		*p*-Value
			Lower Limit	Upper Limit	
UO0–6	−0.154	0.858	0.645	1.140	0.290
SAPS 3	0.068	1.070	1.032	1.110	<0.001
UO6–12	−0.251	0.778	0.567	1.068	0.121
SAPS 3	0.066	1.068	1.030	1.107	<0.001
UO12–24	−0.161	0.852	0.622	1.165	0.316
SAPS 3	0.067	1.070	1.032	1.109	<0.001
UO0–24	−0.174	0.840	0.613	1.153	0.281
SAPS 3	0.067	1.070	1.032	1.109	<0.001

**Table 8:** AUCs for SAPS 3 alone and in multivariate combination with each urinary output parameter in relation to 30-day mortality. Transformed urinary output parameters applied. Stated *p*-values refer to the AUC difference in comparison to the reference line at 0.5. Abbreviations: AUC = area under the curve; 95% CI = 95% confidence interval; UO0–6 = urinary output 0–6 h in mL/h; UO6–12 = urinary output 6–12 h in mL/h; UO12–24 = urinary output 12–24 h in mL/h; UO0–24 = total urinary output after 24 h in mL/h; SAPS 3 = Simplified Acute Physiology Score 3.

	AUC	95% CI		*p*-Value
		Lower Limit	Upper Limit	
SAPS 3 + UO0–6 multivariate	0.766	0.668	0.863	<0.001
SAPS 3 + UO6–12 multivariate	0.779	0.684	0.875	<0.001
SAPS 3 + UO12–24 multivariate	0.768	0.669	0.866	<0.001
SAPS 3 + UO0–24 multivariate	0.770	0.672	0.867	<0.001
SAPS 3	0.765	0.665	0.865	<0.001

## Data Availability

The data that support the findings of this study are not openly available due to reasons of sensitivity but are available from the corresponding author upon reasonable request.
